# Are Silver Nanoparticles Useful for Treating Second-Degree Burns? An Experimental Study in Rats

**DOI:** 10.34172/apb.2021.014

**Published:** 2020-11-07

**Authors:** Débora Antunes Neto Moreno, Mirela Santos Saladini, Fabia Judice Marques Viroel, Murilo Melo Juste Dini, Thaisa Borim Pickler, Jorge Amaral Filho, Carolina Alves dos Santos, Valquíria Miwa Hanai-Yoshida, Denise Grotto, Marli Gerenutti, Stephen Hyslop, Yoko Oshima-Franco

**Affiliations:** ^1^Graduate Program in Pharmaceutical Sciences, University of Sorocaba (UNISO), Sorocaba, SP, Brazil.; ^2^Veterinary Medicine Graduate Course, University of Sorocaba (UNISO), Sorocaba, SP, Brazil.; ^3^Laboratory for Toxicological Research (Lapetox), University of Sorocaba (UNISO), Sorocaba, SP, Brazil.; ^4^Graduate Program in Environmental and Technological Processes, University of Sorocaba (UNISO), Sorocaba, SP, Brazil.; ^5^School of Medical Sciences of the Pontifical Catholic University of São Paulo (PUC-SP), Sorocaba, SP, Brazil.; ^6^Department of Pharmacology, Faculty of Medical Sciences, State University of Campinas (UNICAMP), Campinas, SP, Brazil.

**Keywords:** Hematological parameters, Oxidative stress, Second-degree burn, Silver nanoparticles, Wound healing

## Abstract

***Purpose:*** In this work, the potential usefulness of silver nanoparticles (AgNPs) for treating burn wounds was examined.

***Methods:*** Second-degree burns were induced in male Wistar rats by touching the skin with a heated (70°C) metallic device for 10 s, after which the animals were randomly allocated to one of two groups: control (n=8, treated with sterile saline) and experimental (n=8, treated with AgNPs, 0.081 mg/mL; 50 µL applied to the burn surface). Seven, 14, 21 and 28 days after lesion induction two rats from each group were killed and blood samples were collected for a complete blood count and to assess oxidative stress. The livers were examined macroscopically and skin samples were collected for histological analysis.

***Results:*** Macroscopically, wound healing and skin remodeling in the experimental group were similar to the saline-treated rats. Likewise, there were no significant differences in the histological parameters between the two groups. However, treatment with AgNPs caused a persistent reduction in white blood cell (WBC) counts throughout the experiment, whereas platelet counts increased on days 7 and 28 but decreased on days 14 and 21; there was also an increase in the blood concentration of reduced glutathione on day 7 followed by a decrease on days 21 and 28. There were no significant changes in blood glutathione peroxidase (GSH-Px) and catalase (CAT) activities or in the serum concentration of thiobarbituric acid reactive substances.

***Conclusion:*** The findings of this study raise questions about the potential transitory effects of AgNPs based on the changes in WBC and platelet counts, blood glutathione concentrations and macroscopic hepatic alterations.

## Introduction

Burns are one of the most devastating injuries that directly impact public health systems^1^ and their treatment remains a challenge in health care. Burn wounds caused by contact with thermal (scalding – wet heat; flame – dry heat) and non-thermal (electrical, chemical, cold and radiation) sources result in local (zones of coagulation, stasis and hyperemia)^2^ and systemic (cardiovascular, respiratory, metabolic and immunological) alterations.^3^

Yoshino et al^4^ provided a comprehensive summary of burn terminology, as follows: (1) First-degree burn – cures without scars. (2) Second-degree burn – consists of two types: (a) Superficial dermal burn– a burn that forms a blister in which the dermis on the floor of the blister is red; these burns usually heal 1-2 weeks after epithelialization (epithelial cell migration, proliferation and differentiation) and generally leave no hypertrophic scar. (b) Deep dermal burn– a burn that forms a blister in which the dermis on the floor of the blister is white and anemic; this injury requires 3-4 weeks to heal by epithelialization and is likely to leave a hypertrophic scar or cicatricial keloid. (3) Third-degree burn – a deep burn causing necrosis that involves the full thickness of the skin.

Multiple mechanisms such as vasoconstriction/vasodilation, oxidative stress, hypoperfusion and microthrombosis related to activation of the inflammatory cascade and cell death are involved in burn injury.^5^ These injuries often require complex and expensive treatment by health systems.^6,7^ Protocols for treating burns include combinations of drugs with different mechanisms of action, such as analgesics, non-steroidal anti-inflammatory agents, anesthetics, opioids^8^ and ketamine,^9,10^ in addition to antidepressants and anticonvulsants.^7^ Non-conventional approaches for managing burn-associated pain include the use of heparin that attenuates pain and prevents scarring and contractures.^6^ Complications associated with burns include infection of the wound itself or skin graft donor sites and bacterial contamination of indwelling vascular lines or catheters.^11^

In second-degree burns, the lesion site becomes red, blistered and may be swollen and painful,^12^ making the lesions susceptible to bacterial infection. Silver-based compounds have been used as antimicrobial agents for the treatment of gonococcal infections since the 19^th^ century^13^ and, more recently, silver nanoparticles (AgNPs) have been used to treat bacterial infections and other conditions.^14^ The advantage of using AgNPs to treat microbial infections is their broad spectrum of action that makes the development of antimicrobial resistance more difficult.^15,16^ In addition to their antimicrobial activity, the ability of AgNPs to promote wound healing has also been studied.^17^ However, the potential toxicity and safety issues related to the use of AgNPs still require clarification.^15^

Based on the hypothesis that smaller particles (<10 nm) can induce greater toxicity,^18^ the aim of this work was to examine the ability of AgNPs with a diameter of ~50 nm to promote the healing of second-degree burns induced by thermal contact in rats over a period of 28 days post-injury. This analysis was done by monitoring weekly the changes in a variety of hematological and biochemical (oxidative stress) parameters and by histological analysis of the affected tissue.

## Materials and Methods

### Silver nanoparticle preparation

Two hundred and fifty milliliters of deionized water was added to 45 mg of silver nitrate (AgNO_3_) and 135 mg of polyvinylpyrrolidone in a reactor at 80°C. The silver nitrate in solution was reduced by adding an aqueous solution of 1% sodium citrate to give a silver nitrate:sodium citrate molar ratio of 1:0.68. After 50 min of reaction, the process was terminated and the resulting nanoparticles were characterized as described by Santos et al.^16^ The mean size of the resulting nanoparticles was ~50 nm.

### Animals

Male adult Wistar rats (*Rattus norvegicus* ; 200-250 g) obtained from the Central Animal House of the Institute of Biomedical Sciences of the University of São Paulo (USP, São Paulo, SP) were housed in the Laboratory for Toxicological Research (Lapetox, University of Sorocaba – UNISO, Sorocaba, SP) at 21 ± 2°C and 50 ± 5% humidity on a 12 h light/dark cycle (lights on at 6 AM), with free access to standard rodent chow (Nuvital^®^) and water. The rats were housed 1/polypropylene cage on a wood shaving substrate in ventilated stands (Alesco^®^, Monte Mor, SP, Brazil). The animal protocols were approved by an institutional Committee for Ethics in Animal Use at UNISO (CEUA/UNISO, protocol no. 065/2016) and were done in accordance with current Brazilian legislation (Federal Law no. 11,794, of October 8, 2008), in conjunction with the guidelines for animal experiments established by the Brazilian National Council for the Control of Animal Experimentation (CONCEA) and ARRIVE (Animal Research: Reporting of *In Vivo* Experiments).^19,20^

### Induction and treatment of second-degree burns

Prior to inducing burn lesions, the rats were randomly allocated to one of two groups (n=8 each) that were subsequently treated with either 50 µL of 0.9% sterile saline (saline control, C) or 50 µL of AgNPs (experimental group, E) after lesion induction. Burns were induced based on the method of Walker and Mason^21^ as described by de Campos et al,^22^ but instead of scalding, thermal contact with a cylindrical metallic bar heated to 70°C in a waterbath^23^ was used to allow greater delimitation and standardization of the second-degree lesion. To induce the burn lesion, the rats were anesthetized with a mixture of ketamine hydrochloride (10 mg/kg, i.p.) and xylazine hydrochloride (6 mg/kg, i.p.) and the back then shaved (area: 3 cm × 3 cm) followed by thermal contact for 10 s to induce a 0.6 cm diameter lesion.^24^ Thirty minutes after lesion induction, the rats were treated with sterile saline or AgNPs and then daily for 28 days by placing the desired solution onto the lesion at the same intervals and by the same person (see Supplementary file 1 for video S1 showing the experimental procedure).

For short-term analgesia, the rats received dipyrone (Cifarma Científica Farmacêutica^®^, Goiania, GO, Brazil) in the drinking water for 4 days after lesion induction. The amount of dipyrone added to the water (4 drops/500 mL) was based on a daily water consumption for rats of 10-20 mL water/day,^25^ although the actual consumption in this study was 5-10 mL/rat/day. The changes in body weight and the status of the wounds were recorded daily (see Supplementary file 2, Table S1). The lesions were examined macroscopically and were scored based on the following criteria: 0 – severe infection/extensive necrosis, 1 – moderate exudation/hyperemia, 2 – signs of inflammation/infection at the edges, 3 – initial epithelialization, 4 – partial epithelialization/absence of necrosis, and 5 – epithelialization/hair growth.

Two rats from each group (C and E) were killed with an overdose of ketamine hydrochloride (148 mg/kg, i.p.)^26^ on the 7^th^ (C_7_ and E_7_), 14^th^ (C_14_ and E_14_), 21^st^ (C_21_ and E_21_) and 28^th^ (C_28_ and E_28_) day after lesion induction and blood was collected to assess hematological parameters and indicators of oxidative stress; tissue samples were collected from the burn lesion for histological evaluation.

### Hematological parameters

Blood collected from the posterior vena cava into tubes containing 5 mM ethylenediaminetetraacetic acid (EDTA, disodium salt dihydrate) was used for the hematological analyses.

### Assessment of oxidative stress 

Glutathione (GSH) was determined in total blood by the quantification of sulfhydryl (SH) using the Ellman method.^27^ Glutathione peroxidase (GSH-Px) activity was assayed in total blood according to Paglia and Valentine.^28^ Blood catalase (CAT) activity was assayed according to Aebi.^29^ Lipid peroxidation was assessed by quantifying thiobarbituric acid reactive substances (TBARS) in plasma.^30^

### Histological analysis 

Skin samples from the burn lesion were examined histologically as described by de Campos et al.^22^ Serial sections 5 μm thick were deparaffinized and cleared in xylol before staining with hematoxylin and eosin (HE, 2 sections/animal), Masson’s trichrome (2 sections/animal) or orcein (2 sections/animal).^31,32^ Qualitative histological analysis was done using a Nikon Alphaphot YS-2 microscope and images from the control (C) and experimental (E) groups (n=2 each) were captured with a Nikon E960 Coolpix camera to allow comparison of both groups. In each section, all of the skin layers, from the outer surface to the deepest inner layers, were examined microscopically to assess parameters that included epithelialization, extent of healing, presence of a chronic inflammatory infiltrate, neovascularization, fibroblast proliferation and collagen deposition.^22^

### Statistical analysis

Quantitative data were expressed as the mean ± SEM of the number of rats used in each analysis. Bartlett’s test was used to assess the homogeneity of the data prior to statistical analysis. Since the results of the test confirmed the homoscedasticity of the data, only parametric statistical tests were used in subsequent analyses. Specifically, Student’s unpaired *t* test was used for comparisons between two groups and one-way ANOVA followed by the Tukey-Kramer multiple range test was used for comparisons involving three or more groups. In all cases, the level of significance was set at *P* <0.05. All data analyses were done using InStat (GraphPad Inc., San Diego, CA, USA).

## Results and Discussion

**Figure 1:**
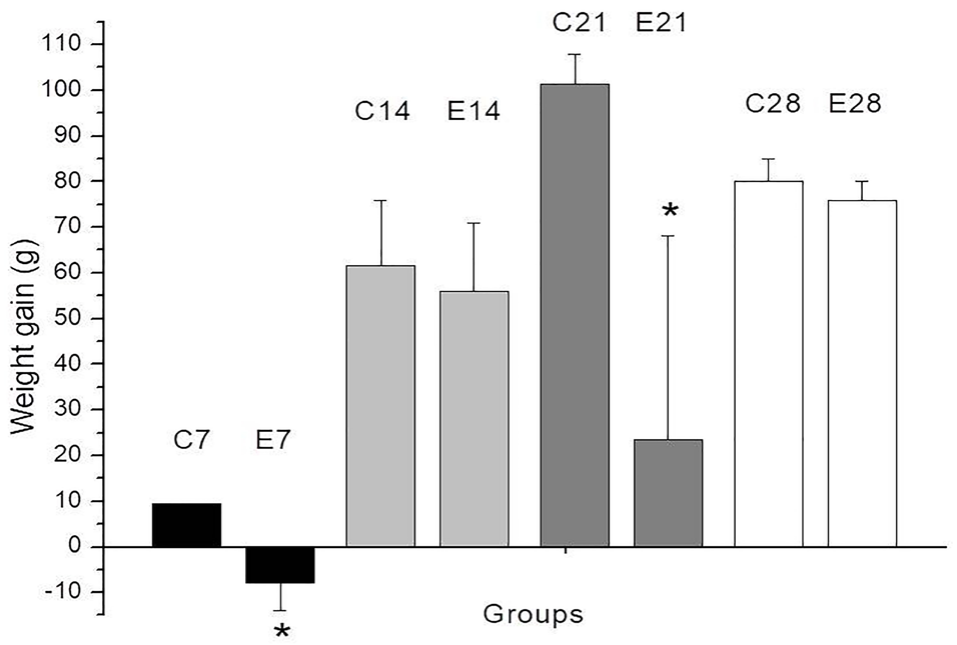
Weight gain (in g) in rats 7, 14, 21 and 28 days after undergoing second-degree burns. The rats in experimental groups E7 and E21 showed weight loss in the first and third weeks compared to the corresponding control groups (C7 and C21, respectively). See the Results/Discussion for an explanation of this weight loss. The columns are the mean ± SEM (n=2/group). *P <0.05 compared to the corresponding control group.

Figure 1 shows the weight gain (in g) on days 7, 14, 21 and 28 after lesion induction. Daily body weight measurements (Table S1) showed a decrease in body weight in both groups of rats (control and experimental) in the first four days following lesion induction. Rat E5 lost weight (21 g by day 21), whereas rat E6 gained weight (68 g in the same period); this variation contributed to the large SEM in this group on day 21. The weight loss in rat E5 may have been caused by contact of the AgNPs with the wound since there was a spontaneous loss of skin from the wound on day 17.

Weight loss in adult animals has been used as an indicator of postoperative pain^33^ but may also reflect the physiological effects of anesthetic or analgesic drugs.^34^ In the first four days after lesion induction, both groups of rats (control and experimental) showed a decrease in body weight. This weight loss was possibly related to the unpleasant taste of dipyrone in the drinking water that may have reduced the rats’ intake of liquid (Table S1). Indeed, when dipyrone-containing drinking water was replaced with fresh water, rats in the control group (burn lesion only) recovered their initial weight and gained around 10 g in three days, i.e., by day 7 (Figure 1), whereas rats in the experimental group (lesion + AgNPs) showed no weight gain; the latter response may reflect a non-specific effect of the nanoparticles. In favor of the latter explanation, rat E5, which showed weight loss (323 g at the beginning – 302 at the end of the experiment), inadvertently tore the skin off the wound and AgNPs came into contact with the wound again.

**Figure 2:**
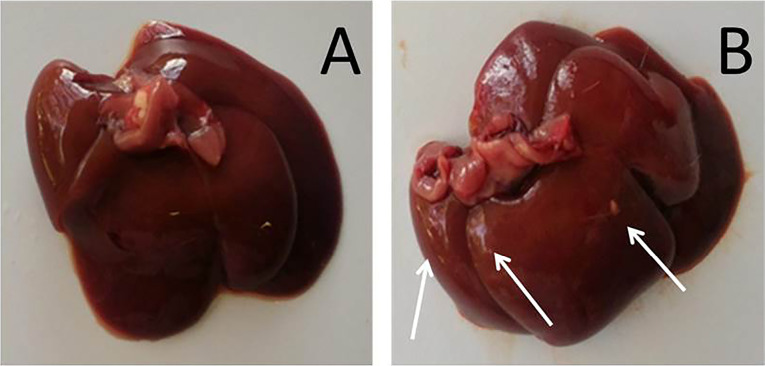
Macroscopic comparison of livers from saline-treated (control; A) and AgNP-treated (experimental; B) rats 7 days after lesion induction. Note the bulky appearance and yellow pigmentation (arrows) of liver from the AgNP-treated rat.

Figure 2 shows the macroscopic appearance of livers from saline-treated (control) and AgNP-treated (experimental) rats on the 7^th^ day after lesion induction. The organs from experimental animals showed changes in size (a more bulky appearance), color (a discolored appearance and foci of yellow pigmentation) and texture (more rigid than control livers). There was no significant difference in liver weight between the groups. The transitory macroscopic effects in the liver reflected the ability of metallic nanoparticles in general to penetrate damaged skin and enter the circulation (possibly via the lymphatic system), eventually accumulating in the liver,^35^ the main organ for detoxification in vertebrates.^36^ The major target organs for AgNP accumulation are the liver and spleen and this deposition may result in metabolic changes, such as switching from glycogenolysis and lipid storage to glycogenesis and lipolysis.^37^ Other effects include a decrease in cell survival, the production of reactive oxygen species, mitochondrial damage, DNA cleavage, autophagy, pyroptosis, apoptosis and necrosis.^18,35^

**Figure 3:**
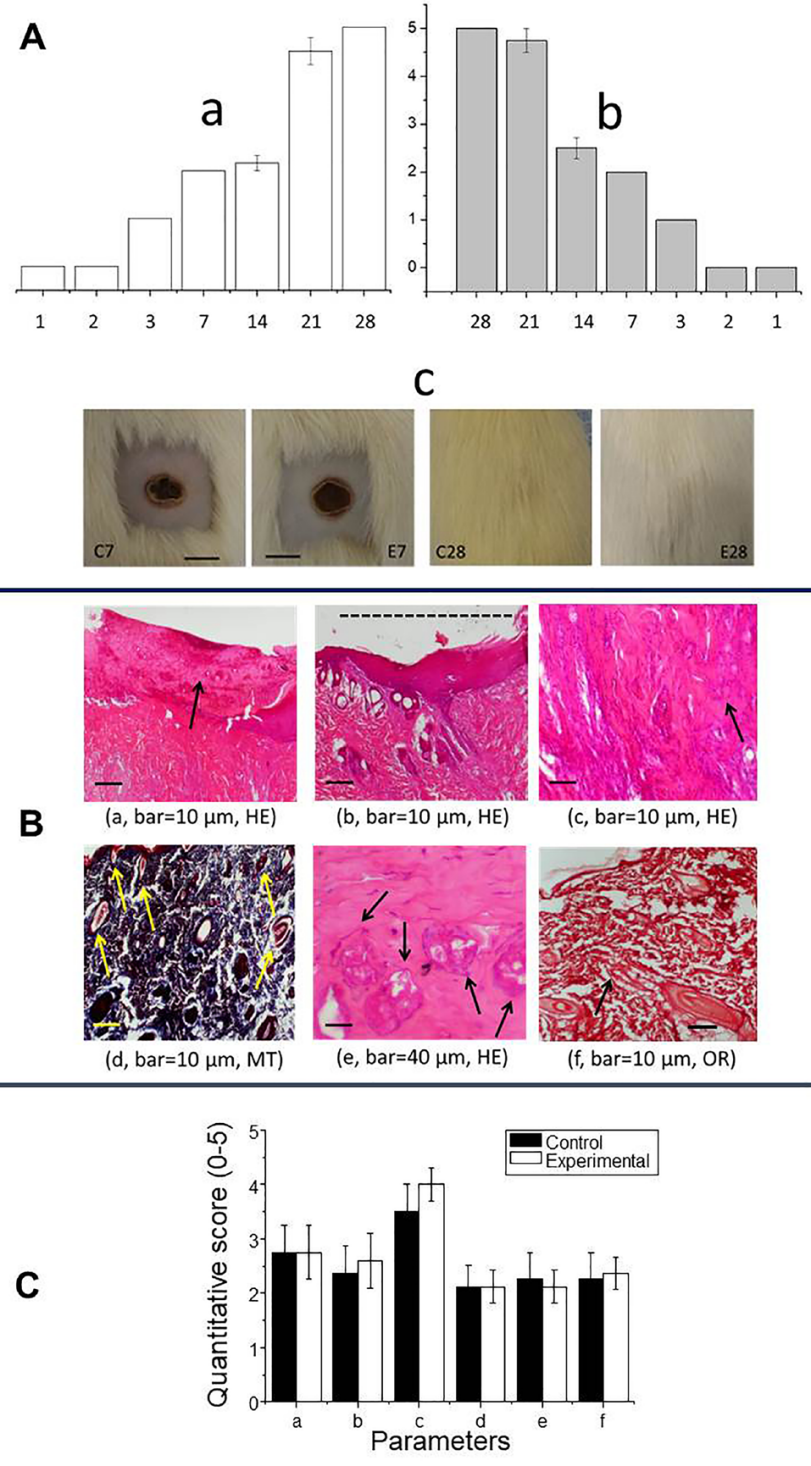
(A) Burn-induced lesions assessed macroscopically in control rats (a, white columns) and AgNP-treated rats (b, grey columns), with the Y-axis showing the lesion scores (from 0 to 5); c, Comparison of the skin healing process seven and 28 days after lesion induction in saline-treated (control; C7 and C28) and AgNp-treated (E7 and E28) rats. Scale bar in C7 and E7: 1 cm. This bar also applies to C28 and E28. (B and C) Qualitative and quantitative histological analysis, respectively. In both panels: a – epithelialization, b – wound healing indicated by the horizontal bar (dashed line), c – chronic inflammatory infiltrate, d – neovascularization, e – fibroblast proliferation and f – collagen deposition. HE, hematoxylin-eosin. MT, Masson’s trichrome. OR, orcein. Arrows in subpanels a and c-f indicate the alteration corresponding to the parameter analyzed; the alteration in subpanel b is indicated by the dashed line. In C, the columns represent the mean ± SEM (n=2/group).

Burn wound healing involves coagulation and hemostasis, inflammation, cell proliferation and remodeling,^38^ as shown in this study through macroscopic and microscopic analyses. Macroscopic examination revealed severe infection and extensive necrosis in the lesions in the first two days after induction (Figure 3A, a and b). The inflammatory response occurred soon after injury and was followed by tissue necrosis, whereas the proliferative phase involved epithelialization from the wound edge to resurface the defect.^39^ The progression of inflammatory signs, epithelialization and hair growth was similar in both groups (there were no differences in the scores for these parameters at any time interval after lesion induction). Dorsal skin images obtained 7 days and 28 days after lesion induction (Figure 3A, c) showed similar healing at both intervals. The demarcation line seen on day 7 consisted of polymorphonuclear cells, whereas hairs were seen covering the lesion by day 28.

In this study, the microscopic pattern of wound healing in the control and AgNP-treated groups was assessed using the parameters described by de Campos et al,^22^ namely, (a) epithelialization, (b) extent of healing based on the lateral extension, (c) presence of a chronic inflammatory infiltrate, (d) neovascularization, (e) fibroblast proliferation and (f) collagen deposition. These parameters, shown in Figure 3B (a-f), were scored on a scale of 0 to 5 to obtain a quantitative assessment (Figure 3C). The lack of infection was corroborated by histological analyses using three stains (hematoxylin-eosin – HE, Masson’s trichome – MT and orcein – OR) to highlight these parameters (Figure 3B and 3C). Collagen is essential for correct tissue repair and the remodeling of wound skin.^40^ Elastic fibers and collagen occur in the reticular dermis, but Masson´s trichrome does not distinguish between these two types of fibers, hence the need to use orcein, which stains elastic fibers a brownish black.^41^ The various parameters (a-f listed above) were observed in the control and AgNP-treated groups, but there were no differences in their frequencies between the two groups (Figure 3C).

Table 1 shows the results of the complete hemogram in the control and experimental groups. There was no difference in the red blood cell (RBC) counts between the two groups, but there was a significant difference in the platelet (PC) and white blood cell (WBC) counts. There were important changes in the WBC counts at all-time intervals in response to treatment with AgNP for 28 days; these changes resulted in leucopenia, neutropenia and lymphocytopenia. Eosinophils showed an increase on the 7^th^ day, a decrease on the 14^th^ day and normalization on the 21^st^ and 28^th^ days.

**Table 1:** Complete blood cell counts for saline-treated (control, C) and AgNP-treated (experimental, E) rats

**Parameter**		**C**_7_	**E**_7_	**C**_14_	**E**_14_	**C**_21_	**E**_21_	**C**_28_	**E**_28_
RBC (10^6^/µL)		6.97 ± 0.21	7.23 ± 0.31	6.94 ± 1.24	6.53 ± 0.47	7.24 ± 0.71	6.49 ± 0.02	7.30 ± 0.19	7.24 ± 0.36
Hb (g/dL)		13.25 ± 0.49	14.35 ± 0.64	13.50 ± 1.70	12.80 ± 0.28	13.80 ± 0.57	12.35 ± 0.07	13.83 ± 0.32	13.60 ± 0.53
Ht (%)		40.00 ± 1.56	43.85 ± 0.07	41.15 ± 5.59	38.90 ± 0.14	43.05 ± 1.77	38.80 ± 0.00	42.00 ± 1.18	40.50 ± 1.73
MCV (femtoliter)		57.50 ± 3.96	60.70 ± 2.69	59.55 ± 2.62	59.75 ± 4.45	59.60 ± 3.39	59.85 ± 0.21	57.63 ± 3.00	55.97 ± 2.15
MCH (pg/cell)		19.00 ± 1.27	19.85 ± 0.07	19.55 ± 1.06	19.65 ± 0.92	19.10 ± 1.13	19.05 ± 0.21	18.97 ± 0.86	18.77 ± 0.60
MCHC (g/dL)		33.15 ± 0.07	32.75 ± 1.48	32.85 ± 0.35	32.90 ± 0.85	32.10 ± 0.00	31.85 ± 0.21	32.93 ± 0.42	33.57 ± 0.21
PC (10^3^/µL)		592.50 ± 292.04	666.00 ± 178.19*	412.50 ± 248.19	229.50 ± 225.57*	869.00 ± 208.89	576.00 ± 183.85*	553.33 ± 230.15	658.67 ± 136.72*
WBC (10^3^/µL)	Leukocytes	8.76 ± 1.81	5.31 ± 0.13*	7.92 ± 0.18	3.33 ± 1.39*	8.09 ± 0.57	2.67 ± 0.11*	6.57 ± 1.07	4.96 ± 0.33*
	Neutrophils	0.88 ± 0.59	0.09 ± 0.09*	0.39 ± 0.11	0.34 ± 0.17*	0.81 ± 0.62	0.07 ± 0.04*	0.67 ± 0,39	0.34 ± 0.17*
	Lymphocytes	6.87 ± 1.32	4.17 ± 0.54*	6.26 ± 0.53	2.43 ± 0.77*	6.57 ± 0.69	2.10 ± 0.10*	5.22 ± 1.02	4.05 ± 0.38*
	Monocytes	0.28 ± 0.11	0.24 ± 0.32*	0.37 ± 0.23	0.10 ± 0.02*	0.19 ± 0.01	0.07 ± 0.03*	0.15 ± 0.13	0.06 ± 0.05*
	Eosinophils	0.04 ± 0.00	0.05 ± 0.00*	0.04 ± 0.00	0.02 ± 0.00*	0.04 ± 0.04	0.03 ± 0.01	0.03 ± 0.03	0.03 ± 0.02
	Basophils	0.70 ± 0.98	0.77 ± 0.18*	0.87 ± 0.23	0.45 ± 0.47*	0.49 ± 0.69	0.40 ± 0.07*	0.50 ± 0.50	0.47 ± 0.29*

Hb, hemoglobin; Ht, hematocrit; MCH, mean corpuscular hemoglobin; MCV, mean corpuscular volume; MCHC, mean corpuscular hemoglobin concentration; PC, platelet count; RBC, red blood cell; WBC, white blood cell. *P <0.05 compared to the corresponding control. The values are the mean ± SEM of N=8rats/group.

A complete blood count is an efficient and simple test that can help in burn assessment. Platelet activation at sites of tissue injury is an important component in the inflammatory response^42^ and could explain the alterations in platelet counts seen in AgNP-treated rats. The initial increase in platelet activation (at E_7_) was suggestive of platelet recruitment to the site of injury and was followed by a decrease at E_14_ and E_21_. WBCs are involved in inflammatory and immune responses^43^ and the alterations seen in the complete blood cell counts were indicative of healing of the burn lesion since a decrease in circulating WBCs reflects the recruitment of these cells to sites of severe tissue damage. The changes in cell numbers seen in AgNP-treated rats suggested that these cells may consider the nanoparticles to be foreign material. Our results agree with a previous study that reported chronic inflammation after exposure to AgNPs.^44^

**Figure 4:**
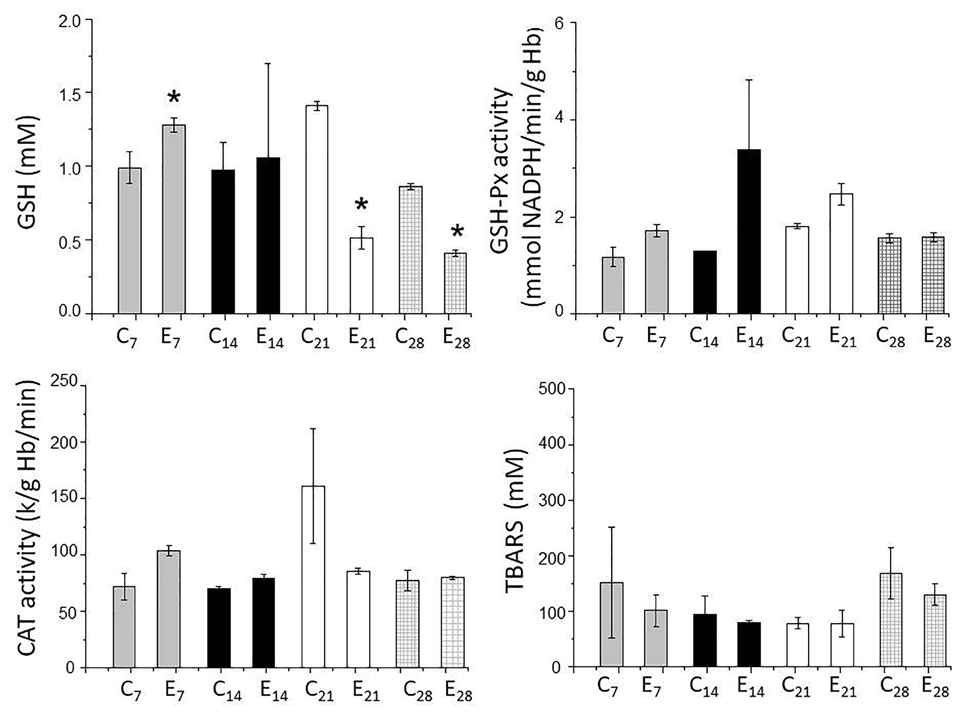
Indicators of oxidative stress in rats with burn lesions treated with sterile saline (C – control) and burn lesions treated with AgNP (E – experimental). CAT – catalase, GSH – glutathione, GSH-Px – glutathione peroxidase and TBARS – thiobarbituric acid reactive substances. Blood samples for assessing oxidative stress were collected from rats killed 7, 14, 21 and 28 days after lesion induction. Note the initial increase in GSH (day 7) and subsequent decrease (days 21 and 28). The columns represent the mean ± SEM (n=2/group). *P <0.05 compared to the corresponding control.

Figure 4 shows the changes in the parameters for oxidative stress. There was an increase in blood GSH levels in the first week, but no changes in the other parameters (GSH-Px, CAT and TBARS) compared to the control group. The increase in the levels of GSH in the first week was suggestive of an inflammatory event since GSH, the most-important redox regulator, controls inflammatory processes.^45^ Since there were no significant differences in GSH-Px between the two groups, the increase in GSH may reflect the action of GSH-reductase that converts the disulfide form (GSSG) to GSH.

In contrast to the findings for the first week, a significant decrease in GSH levels was detected on the 21^st^ and 28^th^ days in AgNP-treated rats and may reflect a continued effect of AgNPs. Since the GSH-Px activity was unaltered in both groups, the redox system was clearly disabled. GSH-Px converts circulating GSH to GSSG in tissues, thereby contributing to the compartmentalized homeostasis of GSH/GSSG.^46^ Based on this action, it is possible that cleavage of the covalent bonds of GSH by AgNPs could lead to a decrease in the circulating levels of this compound. As a consequence of this depletion there would be less cellular protection against apoptosis,^47^ with pro- and antiapoptotic pathways being affected. The cytotoxicity of AgNPs may involve mitochondrial pathways that reduce GSH. Since there was little absorption of AgNPs on days 21 and 28 when healing was at an advanced stage, the decrease in GSH content seen at these intervals could be continued.

CAT activity, an indicator of environmental stress,^48^ did not differ between the groups, indicating that reactive oxygen species, including H_2_O_2_, were not generated after exposure to nanoparticles. The lack of significant changes in TBARS levels indicated that the nanoparticles did not induce lipid peroxidation.

## Conclusion

Although previous reports have described the beneficial effects of AgNPs in neutralizing the neurotoxicity of *Bothrops jararacussu* snake venom in mouse phrenic nerve-diaphragm preparations *in vitro*^49^ and Silva et al.^50^ concluded (based on a systematic review) that dressings containing nanocomposites, including AgNPs, are quite promising for promoting wound healing, no such advantages were seen in this study. In particular, the findings reported here, particularly in relation to the changes in WBC and platelet counts, GSH levels and hepatic alterations, raise questions as to the therapeutic efficacy of AgNPs, especially when applied directly to second-degree burns. These findings suggest the need for caution in the clinical use of AgNPs for wound healing.

## Ethical Issues

The animal protocols described in this work were approved by an institutional Committee for Ethics in Animal Use and were done in accordance with current Brazilian legislation and international guidelines, as described in the Methods.

## Conflict of Interest

The authors declare that they have no competing interests.

## Acknowledgements

This work was supported by Financiadora de Estudos e Pesquisa (FINEP, grant no. 07/2010), Fundação de Amparo à Pesquisa do Estado de São Paulo (FAPESP, grant nos. 2004/09705-8, 2007/53883-6, 2008/52643-4, 2008/11005-5, 2012/08271-0, 2015/01420-9 and 2016/12599-2) and CAPES/Prosup and Probic/Uniso.

## Supplementary Materials

Supplementary file 1 contains Video S1.

Supplementary file 2 contains Table S1.
