# Supplementary material for: Are Silver Nanoparticles Useful for Treating Second-Degree Burns? An Experimental Study in Rats
Source: Adv Pharm Bull. 2020 Nov 7;11(1):130–6. doi: 10.34172/apb.2021.014 (PMC7961217; doi:10.34172/apb.2021.014)
Supplement: Supplementary file 2 — contains Table S1. [file apb-11-130-s002.pdf]

Table S1. Daily body weight measurements (g) for control (C) and experimental (E) rats.

C or E	Body weight (g) in relation to days after burn injury																											
	0	1	2	3	4	5	6	7	8	9	10	11	12	13	14	15	16	17	18	19	20	21	22	23	24	25	26	27	28
C1	227	215	213	197	191	221	226	237	-	-	-	-	-	-	-	-	-	-	-	-	-	-	-	-	-	-	-	-	-
C2	265	246	254	236	224	263	266	274	-	-	-	-	-	-	-	-	-	-	-	-	-	-	-	-	-	-	-	-	-
C3	245	230	238	218	210	246	252	261	268	266	274	275	280	283	292	-	-	-	-	-	-	-	-	-	-	-	-	-	-
C4	216	225	234	216	207	240	248	249	256	258	259	262	267	275	292	-	-	-	-	-	-	-	-	-	-	-	-	-	-
C5	208	213	220	207	195	233	240	244	248	259	259	267	269	275	271	280	283	282	288	288	297	303	-	-	-	-	-	-	-
C6	214	215	217	201	193	227	242	243	248	256	260	265	276	282	286	287	291	291	300	302	310	322	-	-	-	-	-	-	-
C7	300	283	271	259	244	280	291	296	297	303	307	315	321	322	330	336	331	338	345	345	350	352	360	356	360	363	366	372	375
C8	230	223	232	216	206	240	241	249	257	257	261	264	271	271	276	283	282	285	296	294	305	301	303	297	303	304	310	309	315
E1	262	245	232	217	200	244	257	260	-	-	-	-	-	-	-	-	-	-	-	-	-	-	-	-	-	-	-	-	-
E2	290	254	236	222	211	250	263	276	-	-	-	-	-	-	-	-	-	-	-	-	-	-	-	-	-	-	-	-	-
E3	225	232	219	211	201	245	243	257	262	259	272	276	281	285	296	-	-	-	-	-	-	-	-	-	-	-	-	-	-
E4	285	258	241	245	235	278	282	286	295	295	300	305	316	311	326	-	-	-	-	-	-	-	-	-	-	-	-	-	-
E5	323	214	216	198	191	228	233	240	244	248	254	261	263	265	270	270	276	277	285	285	295	302	-	-	-	-	-	-	-
E6	260	244	226	215	213	252	259	261	270	273	281	285	296	294	298	307	309	308	311	316	322	328	-	-	-	-	-	-	-
E7	223	213	212	198	189	225	232	235	241	237	246	247	251	255	261	264	264	269	268	272	283	286	285	281	286	285	294	293	304
E8	244	234	224	206	195	234	245	247	252	260	263	265	275	274	277	289	283	289	288	291	305	305	304	307	310	311	311	313	316

Dipyrone in the drinking water (days 1–4) — Fresh water (days 5–7)

C – control (sham) rats, E – experimental (burn injury) rats. The values in green indicate the body weights at the start of the experiment and those in red correspond to the body weights 7, 14, 21 and 28 days after producing the burn injuries (n=2 rats killed at each interval).
